# Automated Objective Routine Examination of Optical Quality of Rigid Endoscopes in a Clinical Setting

**DOI:** 10.1371/journal.pone.0059579

**Published:** 2013-03-28

**Authors:** Rens Wientjes, Herke J. Noordmans, Jerine A. J. van der Eijk, Henk van den Brink

**Affiliations:** University Medical Centre Utrecht, Utrecht, The Netherlands; University of California Irvine, United States of America

## Abstract

Rigid endoscopes degrade during clinical use due to sterilization, ionizing radiation and mechanical forces. Despite visual checks on functionality at the department of sterilization, surgeons are still confronted with suboptimal instruments as it is difficult to assess this degradation in an objective manner. To guarantee that endoscopes have sufficient optical quality for minimal invasive surgery, an experimental opto-electronic test bench has been developed in order to be used at the department of sterilization. Transmission of illumination fibres and lens contrast values are stored in a database to enable empirical criteria to reject endoscope for further clinical usage or to accept endoscopes after repair. Results of the test bench are given for an eight month period, where a trained operator performed 1599 measurements on 46 different types. Stability of the system, trends in quality of clinical endoscopes, and effect of repair or replacement were assessed. Although the period was too short to draw firm conclusions, a slow downwards trend in quality of clinically used endoscopes could be observed. Also, endoscopes generally improve in quality after repair or replacement, while endoscope replacement seems to slightly outperform endoscope repair. To optimize the measurement process, a new system is being developed requiring less user interaction and measuring more optical parameters of an endoscope. By commercializing this system, we hope that measurements at different hospitals will give improved insight which acceptance and rejection criteria to use and which factors (usage, cleaning protocol, and brands) determine the economic lifetime of endoscopes.

## Introduction

Since 1990 endoscopic surgery has become a commonly used technique in a wide range of applications [1]. Two types of endoscopes exist: rigid or flexible. Flexible endoscopes are used to examine the interior of organs (such as oesophagus, stomach, duodenum, colon, lung, etc.). Compared to flexible endoscopes, rigid endoscopes generally provide better image quality for the same diameter and can be sterilized in an autoclave to enable usage in artificially created body cavities. Rigid endoscopes are applied for diagnoses and surgical interventions through small incisions or natural orifices. Although the optical quality of both types of endoscopes is important, we will focus on the optical quality of rigid endoscopes in this paper.

**Figure 1:**
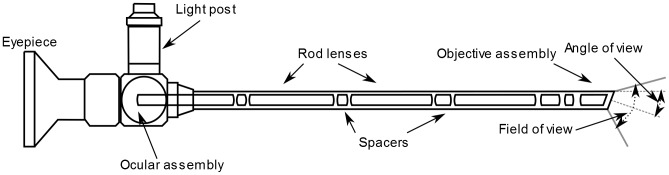
Schematic of a rigid endoscope.

Endoscopic surgery requires more complex and fragile instruments than conventional open surgery. A typical system for endoscopic surgery consists of a bright light source, a light cable, endoscope, a camera, a video processor and a monitor. In this chain, the endoscope is the most complex and fragile critical part as it is can be easily damaged but still is being re-used after cleaning and sterilization (figure 1). Being a small tubular telescope it offers two pathways for light transport. First, the illumination pathway consisting of a fibre bundle transmitting the light into the body cavity illuminating the surgical area. Second, the viewing pathway where several lenses transport the image of the surgical area back to the eyepiece.

Rigid endoscopes degrade during clinical use due to sterilization, ionizing radiation and mechanical forces. The endoscope is fragile and therefore at high risk of damage. The degradation is often difficult to assess by the naked eye [2] [3]. The image as observed by the surgeon is used for diagnosis and Intervention guidance. Poor image quality may increase the risk of wrong diagnosis and treatment. Trouble shooting a defective endoscopic system is a time-consuming and frustrating process. Expensive OR time is lost when parts of the system have to be exchanged to determine which component is malfunctioning [4]. There is a need for a practical quantitative testing method to assure the optical quality of an endoscope for clinical use [2] [3].

### Current Situation

Dutch regulations require a quality control of endoscopes before every reuse [2] [5]. The quality check is performed at the department of central sterilization after the endoscopes have been cleaned and before they are packed for sterilization [2] [4]. Often the examination of the endoscope is limited to a visual check of the endoscope; sometimes cold spray is applied to detect moisture or an additional lens is used to detect impurities at the individual lens surfaces [2] [4]. When endoscopes are found with bad optical quality during surgery or quality control at the department of central sterilization, they are typically sent to the original manufacturer to be replaced by new or refurbished endoscopes. For many years, the original manufacturer was the sole source of endoscope refurbishment. With the growth of minimal invasive surgery, third-party repair companies have been established during the past decades [4], in general offering repairments at lower costs.

A problem with these manual tests at the department of central sterilization is that it is difficult to assess the quality of an endoscope objectively. This makes it hard to maintain clear and uniform acceptance and rejection criteria. Also, for a human observer it is almost impossible to detect gradual changes in optical quality over time, especially for a large number of different types of endoscopes. Some manufacturers of endoscopes do specify acceptance and rejection criteria, like maximum number of dark illumination fibres and check on dents and lens cracks. It is however, unclear what the relation is with visibility problems in the OR and whether these criteria are under- or overestimations.

There are several possibilities to measure the optical quality of endoscopes. The company Karl Storz sells a test bench (type 499T) with one single circular target and a fibre transmission measurement. The result of the image quality is assessed visually. Reference levels for Karl Storz endoscopes are given on a printed paper. This instrument is intended to be used by repair companies and other specialised users. EndoLume from Lighthouse Imaging can measure the light transmission of the illumination fibres. The result is displayed and has to be compared with the values that can be looked up per type of endoscope. The EndoBench, also from Lighthouse Imaging, is capable to measure the optical quality of endoscopes. This system uses slides as targets and is intended for use at the department of medical technology or repair firms. These products require experienced users. Comparison between endoscopes of the same type is not implemented in the software. Unfortunately none of these test instruments are intended for routine use at the department of central sterilization.

### Experimental Test Bench

Our aim was to create a device that is capable of objectively measuring the optical quality at the department of central sterilisation. To minimize workflow disturbance, the quality control(s) should preferably be automatic, quick and easy to perform. Also, it should provide unambiguous outcomes (pass/fail indication) and have an intuitive user interface. In addition to the regular quality-control check, the device should facilitate the acceptance of new or repaired endoscopes.

In the year 2005 we started the design of our first experimental test bench [6]. Based on clinical measurements, several problems were addressed in the years after: Stability of the LCD display, reproducibility of measurements (electronic ruler, camera focus) and switch to serial number identification, because endoscopes were exchanged between baskets during surgery [7] [8].

To overcome these problems, we started a new measurement period from 2011-07-07 to 2012-3-06, where a trained operator from the technical department performed the measurements. Identification was performed on the serial number of an endoscope and repaired and new endoscopes were registered carefully. This paper describes the results of the measurements during this period. In this period, the measurements were performed two times a week, due to the limited availability of the trained operator.

## Methods

### Technical Approach

To assure the optical quality of rigid endoscopes in clinical practice, a test bench was developed. Dedicated software was developed to instruct the operator, generate test patterns, capture images, analyse and store these results.

**Figure 2:**
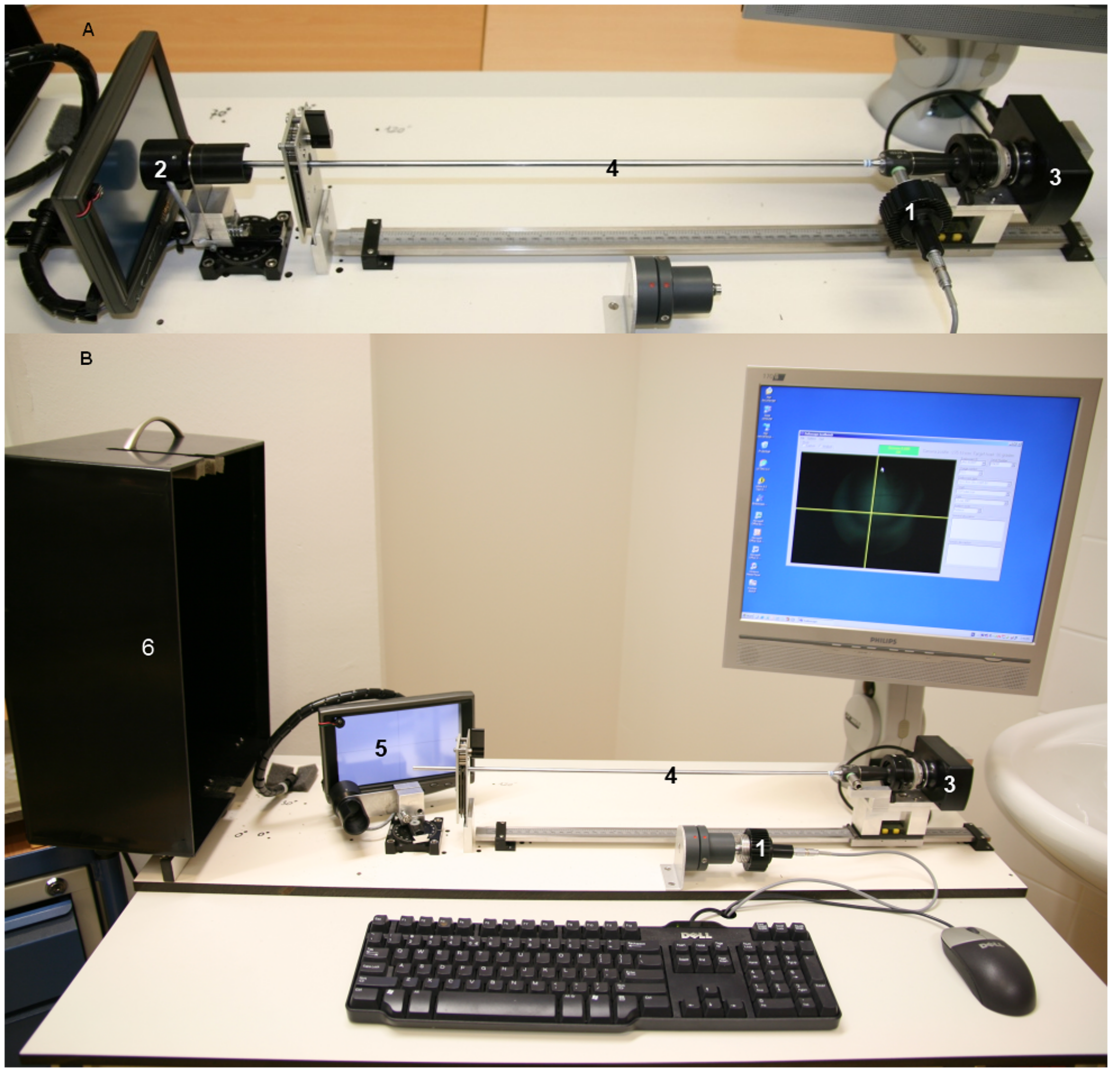
Illustration of measuring the two optical pathways of an endoscope. (A) The transmission of the illumination fibres is measured using a power LED (1) and a photo cell (2). (B) The camera (3) captures a line pattern trough the endoscope (4). The LCD-screen (5) is placed at a 70° angle for this particular endoscope. Surrounding light is blocked with a light cover (6) (open in this picture).

The endoscope was mounted in the endoscope test bench (figure 2) and attached to the camera. Based on the type of endoscope the camera was set to the correct position, within 0.1 mm, measured on an electronic ruler. The screen angle was set in discrete steps to match the angel of view of the type of endoscope under test. The illumination pathway was measured using a white power LED and a photocell. The current output is converted to a voltage and amplified by a low noise differential operational amplifier. The output is sampled by an AD converter and fed back to the PC. The measurement started with a calibration with the light source attached directly to the photocell. Then the amount of transmitted light was measured with the light source coupled to the endoscope’s light-coupling connector and the distal end of the endoscope pointed at the photocell.

**Figure 3:**
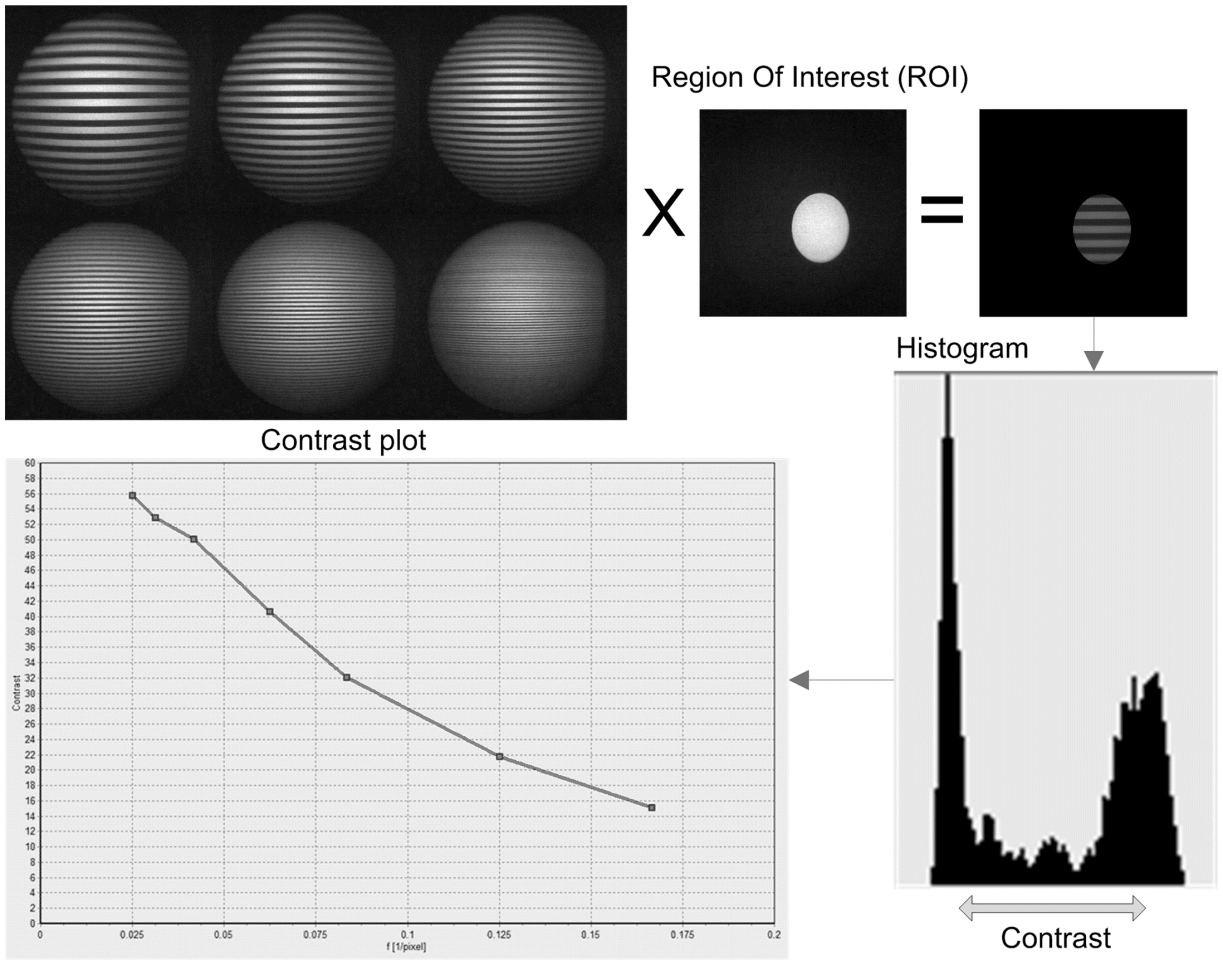
Overview of contrast calculation (clockwise). Test patterns are generated on the LCD display and pictures are captured by a camera through the endoscope. One of the test patterns is a circle in the centre of the LCD display, defining the region of interest where the endoscope image should be sharp. The contrast for a specific line pattern is determined by calculating the distance between the light and dark peak in the histogram for this region of interest.

The viewing pathway was assessed using a computer generated test pattern displayed on a LCD display. The test patterns consisted of alternating lines of black and white with seven different spatial frequencies. The test patterns were captured by a high-resolution camera fitted on the eyepiece of the endoscope. The pixels in the region of interest were binned in grey level. The contrast was defined as the distance in grey levels between 25% and 75% of the accumulated counts of pixels with ascending grey levels. The method is illustrated in figure 3. To allow comparison between successive measurements, the contrast value was averages over the seven line frequencies. After testing the endoscope on the test bench, cold spray is applied to the eye piece of the endoscope te detect moisture. All measurement results were stored in a custom database.

### Acceptance and Rejection Levels

To give the user insight when to accept or reject an endoscope for clinical use, we set acceptance levels before accepting new, refurbished or repaired endoscopes and rejection levels for rejecting clinical used endoscopes before re-use. We decided to base acceptance and rejection levels on the results stored in the test bench’s database: New, refurbished or repaired endoscopes were accepted when their measurement values were at least 40% of the measurement values of the best endoscopes of the same type found in the database. The rejection level was set to 20% of the best endoscope of the same type. Both values were chosen conservatively, in order not to challenge the hospital with high replacement costs in this experimental phase.

### Reference Endoscope

To assess the stability of the system the illumination-fibre transmission and contrast were determined for the reference endoscope. In every measurement session the reference endoscope (a Storz 10320 A bronchoscope) was measured once. The reference endoscope is used only to verify the stability of the system. It is not used, washed or sterilized.

## Results

Over the measurement period, 1599 measurements were performed in the department of central sterilization on 288 rigid endoscopes of 46 different types. Forty-nine of these measurements where performed on the reference endoscope.

### System Stability

**Figure 4:**
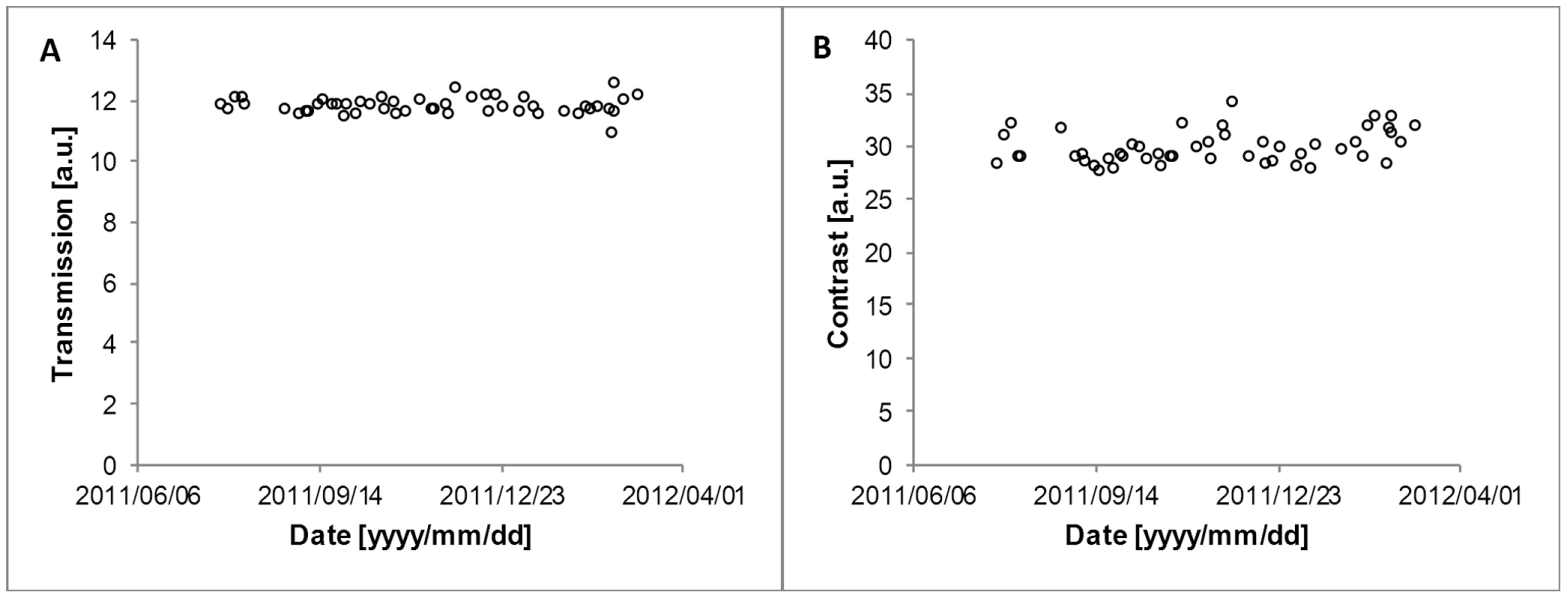
Scatter plot of measurements of the reference endoscope over the measuring period. (A) The illumination fibre transmission is shown. Full scale (100 units) transmission is defined by the value that is measured with the light source directly fitted on the photo cell. (B) The measured contrast is shown.

The illumination-fibre transmission and contrast value of the reference endoscope are shown in figure 4. Ninety-five per cent of the transmission data points are within two per cent of the mean value. The transmission data show no evidence of a drift in this period (figure 4, left). The average contrast value is displayed in figure 4, right. Ninety-five per cent of the contrast data points are within three per cent of the mean value. We therefore considered differences larger than five per cent to be significant for the rest of the measurements.

### Performance Tracking Over Time

**Figure 5:**
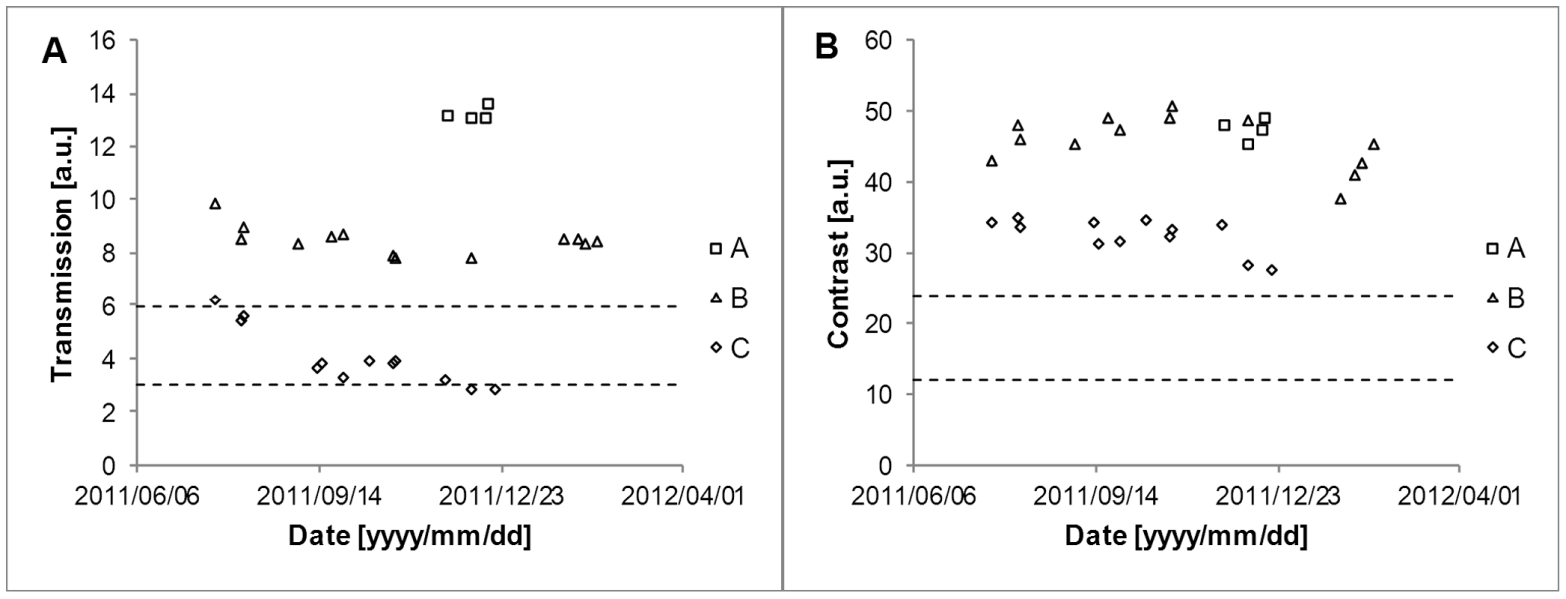
Measurements of illumination fibre transmission (A) and contrast (B) over time for three endoscopes of the same type. Examples of a good (A), moderate (B) and low-performing (C) endoscope are shown. The upper dashed line is the acceptance level for new or repaired endoscopes, the lower dotted line is the rejection level for clinically used endoscopes.

The system allowed us to track the performance of an individual endoscope or a group of endoscopes of the same type over time. Figure 5 shows a typical example of a group of three endoscope of the same type. A slight downwards trend over time was observed, for endoscope C this trend was significant for both the transmission and the contrast. The transmission of one endoscope (C) reached the rejection level and was sent in to be repaired.

### New and Repaired Endoscopes

**Figure 6:**
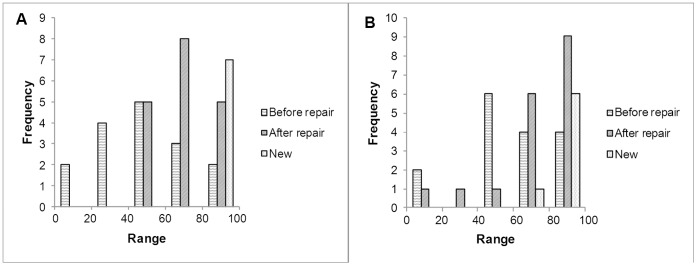
Histogram of relative illumination fibre transmission (A) and contrast (B) of repaired and new endoscopes. The relative performance is calculated. The 100% reference level is set by the best performing endoscope of the same type. The contrast of two endoscopes was below 40% after repair, those two were sent back to the repair company.

In the measurement period, 68 endoscopes were sent in to be repaired or replaced. In sixteen cases the endoscope has been measured before repair, in eighteen cases this was done immediately after repair. In fourteen cases the same endoscope was measured before and after repair. Seven new endoscopes were measured in this period. Six out of seven new endoscopes measured more than 80% compared to the best performing endoscope of the same type. The contrast of one new endoscope was measured at 67% (see figure 6).

A typical example of defects and their frequency of occurrence is shown table 1. Most of the time, more than one deficiency was reported per endoscope. Most repairs were on the lens system. Replacing the illumination fibres is relatively expensive and rarely opted for.

**Table 1:** Identified deficiencies, as reported by repair firm in the eight-month measurement period based on 68 endoscopes that were sent in.

Defect	Counts
Dusty, particles are visible on the lens surface	54
Mechanical damage, dents, scratches, bended shaftor damaged tip	46
Broken rod lens(es)	41
Sapphire window at the tip needs to be replaced	27
Objective assembly needs to be replaced	17
Moisture condenses inside the endoscope	11
Eye piece loose, torn or adhesive damaged	9
Fibre transmission low	5
Other	3

**Figure 7:**
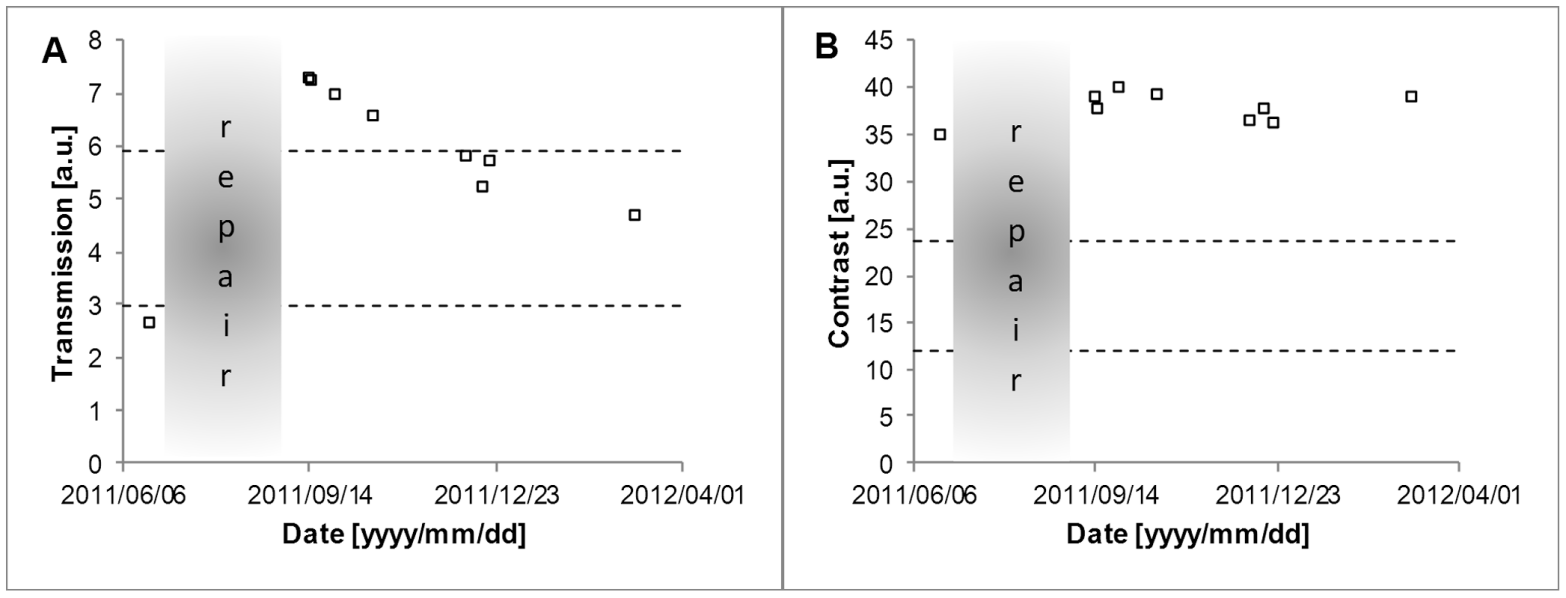
Measurements of illumination fibre transmission (A) and contrast (B) before and after repair for a single endoscope. The endoscope showed mechanical damage at the distal end. The sapphire window was replaced by the repair firm. The upper dashed line is the acceptance level, the lower dashed line is the rejection level. The acceptance and rejection levels were set at respectively 40% and 20% of the measurement values of the best endoscopes of the same type.

In general the performance of a repaired endoscope was better after than before repair. The transmission increased with more than five per cent in nine cases, the remaining five cases stayed within five per cent of the value before repair. In ten cases the contrast improved by more than five per cent, in two cases the contrast decreased more than five per cent. The 100 per cent reference level is set by the best performing endoscope of the same type. In general the performance of a repaired endoscope seems to be somewhat below that of a similar, new endoscope, see figure 6. Figure 7 shows an example of the quality of a repaired endoscope. The transmission and the contrast were improved by the repair. Both measures were above the acceptance level. The transmission drops rapidly in the period after repair.

## Discussion

In this paper we have presented the results of measuring the quality of rigid endoscopes at the department of central sterilization by a trained person from the technical department in an eight months period. Based on the measurements of the reference endoscope, we considered changes above 5% to be significant. We investigated whether the test bench can be used for quality control of clinically used endoscopes by measuring the illumination-fibre transmission and the transferred contrast of line patterns by the lens system.

### Track Performance Over Time

The endoscope test bench is able to track the optical quality of endoscopes which are used clinically. During the endoscope’s lifecycle gradual deterioration is expected. Our measurements seem to agree with that, as a gradual deterioration in both the fibre transmission and the quality of the lens system can be observed. From the current measurements it is difficult to draw firm conclusions. As measurements were performed two times a week, endoscopes could be re-sterilized several times in-between. It is therefore hard to determine which factors cause the fibre transmission to drop rapidly after repair (figure 7). Our hypothesis is that although the fibres at the light post are cleaned and polished at the repair firm, debris from cleaning in combination with a high power light source is probably responsible for these burning-in effects. We expect that, when all endoscopes are measured in every re-use cycle, we can distinguish between a normal and an abnormal slope of deterioration.

During this phase of development we have set the levels for rejection of endoscopes at 20% of the best endoscope of the same type. This value is chosen conservatively as during implementation we did not want to reject large numbers of endoscopes at once, given the fact that repairing or replacing an endoscope takes about four weeks and most departments only have 4–6 endoscopes of the same type. Therefore we started with rejecting the poorest performing endoscopes combined with severe moisture inside. Moisture was detected in 43 out of 288 endoscopes. We detected moisture 150 times, but some surgical departments kept using the leaking endoscopes up to 10 times after we reported the issue. At the time of writing it is not clear whether the procedure to report these defects to the operating room is insufficient, or the severity of the defect is subordinate to the need of the use.

### Measurement Setup

The camera has to be accurately focused and the endoscope has to be positioned within 0.1 mm of the position stored for that type in the database. This requires attentiveness of the operator and can best be performed by technically trained staff. We learned that the next version of an endoscopic test bench should have a more robust user interface and should be less dependent on manually performed actions. The identification of the tested endoscope should be robust and automated to avoid measurement results stored under the wrong identity. During the measurement period reported here, we used a single operator instructed to use serial numbers to minimize this effect.

### Future Development

Based on the experience with our experimental test bench in clinical practice, we believe that there is a clear need for objective quality control of endoscopes. To enable robust quality control of endoscopes, we envision a next generation test bench in which endoscopes are identified automatically, which is easy to use by quick and robust mounting of the endoscope and has a simple user interface with no more than one start button. It should also be connected to the hospital’s track and trace system. A test bench that meets these requirements will bring 100% functional control of rigid endoscopes one step closer.

To get closer to these goals, we started collaborating with DOVIDEQ Medical B.V. (Deventer, The Netherlands) to develop the “Scope Control”. It has been designed for quick and easy use at the department of sterilization. It offers quick mounting of endoscope followed by automatic measurements of transmission of the illumination pathway, and transmission, colour, dust, moisture and view angle of the viewing pathway. Measurements are compared with reference values in a database for objective acceptance or rejection. As the database will be shared among hospitals, deterioration trends and endoscope brands may be compared.

### Conclusions

The opto-electronic test bench has been proven in clinical practice. It is possible to record the performance of rigid endoscopes in a quantitative way. It provides useful information about the current state of the endoscopes in use. Endoscopes can be rejected in time, based on quantitative measurements. Repaired or new endoscopes can be assessed and compared to other endoscopes of the same type before they are accepted.

To optimize the measurement process at the department of sterilization, a new system called ‘Scope Control’ is being developed in collaboration with DOVIDEQ Medical requiring less user interaction and measuring more optical parameters of an endoscope. By commercializing this system, we hope that measurements at different hospitals will give improved insight which acceptance and rejection criteria to use and which factors (usage, cleaning protocol, and brands) determine the economic lifetime of endoscopes.
